# Interplay of weak interactions in the atom-by-atom condensation of xenon within quantum boxes

**DOI:** 10.1038/ncomms7071

**Published:** 2015-01-21

**Authors:** Sylwia Nowakowska, Aneliia Wäckerlin, Shigeki Kawai, Toni Ivas, Jan Nowakowski, Shadi Fatayer, Christian Wäckerlin, Thomas Nijs, Ernst Meyer, Jonas Björk, Meike Stöhr, Lutz H. Gade, Thomas A. Jung

**Affiliations:** 1 Department of Physics, University of Basel, Klingelbergstrasse 82, 4056 Basel, Switzerland; 2 PRESTO, Japan Science and Technology Agency (JST), 4-1-8 Honcho, Kawaguchi, Saitama 332-0012, Japan; 3 Laboratory for Micro- and Nanotechnology, Paul Scherrer Institute, 5232 Villigen PSI, Switzerland; 4 Departamento de Física Aplicada, Instituto de Física Gleb Wataghin, Universidade Estadual de Campinas, Campinas 13083-859, Brazil; 5 Department of Physics, Chemistry and Biology, IFM, Linköping University, Linköping 581 83, Sweden; 6 Zernike Institute for Advanced Materials, University of Groningen, Nijenborgh 4, 9747 AG Groningen, The Netherlands; 7 Anorganisch-Chemisches Institut, Universität Heidelberg, Im Neuenheimer Feld 270, 69120 Heidelberg, Germany

## Abstract

Condensation processes are of key importance in nature and play a fundamental role in chemistry and physics. Owing to size effects at the nanoscale, it is conceptually desired to experimentally probe the dependence of condensate structure on the number of constituents one by one. Here we present an approach to study a condensation process atom-by-atom with the scanning tunnelling microscope, which provides a direct real-space access with atomic precision to the aggregates formed in atomically defined ‘quantum boxes’. Our analysis reveals the subtle interplay of competing directional and nondirectional interactions in the emergence of structure and provides unprecedented input for the structural comparison with quantum mechanical models. This approach focuses on—but is not limited to—the model case of xenon condensation and goes significantly beyond the well-established statistical size analysis of clusters in atomic or molecular beams by mass spectrometry.

## Abstract (web summary)

Condensation in the regime of weakly interactions is of fundamental importance. Here, the authors study the condensation process one atom at a time, showing the forces driving the behaviour of xenon atoms as they condense into aggregate structures in nanoscale pores.

## Graphical abstract

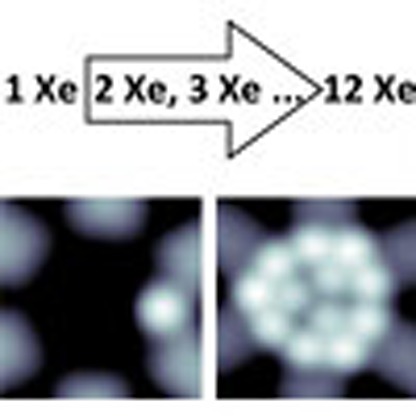

Condensation is a fundamental process, and the interactions involved in the aggregation of atoms or molecules govern the structure of condensates^1^,^2^. Moreover, at the nanoscale level the properties of a condensate depend on its size, structure and bonding between the atoms or molecules. Although the analysis of noble gas condensates of different sizes, interacting by isotropic van der Waals forces, has provided valuable insight into the mechanisms of particle clustering^3^,^4^,^5^,^6^, the condensation in a real environment usually proceeds under the competing influence of weak forces.

In order to take an interplay of such forces into account in a model condensation process, we chose a nanopatterned vacuum/solid interface, which not only provides the competition between interparticle forces and interactions with a surface^7^,^8^,^9^,^10^ but may also give rise to a more complex interplay of forces^11^,^12^,^13^, which, as we observe, governs the emergence of structural patterns. We demonstrate how the atom-by-atom condensation of noble gas atoms proceeds under the influence of competing interactions. This is conveniently probed within the atomically defined cavities of a supramolecular network, generated on the metal substrate. This approach is based on the ability of such on-surface networks to trap different adsorbates and thus to create host–guest systems^14^,^15^,^16^,^17^,^18^,^19^,^20^.

## Results

### Design of the host–guest system

**Figure 1:**
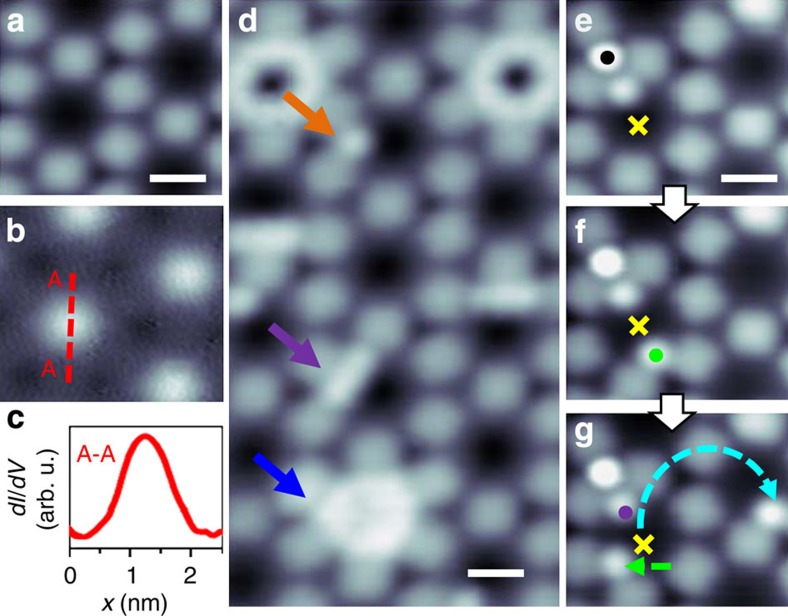
Repulsive interaction between Xe and the electronic quantum well state. (**a**) STM image of the vacant network and (**b**) simultaneously acquired d*I*/d*V* map taken at the energy of the confined state: −200 meV (the bright colour reflects a local density of states maximum). (**c**) The cross-section taken along the red line showing the spatial distribution of the confined state. (**d**) STM image of Xe adsorbed in some pores after exposure to 120 L (Langmuir) of Xe at 9 K: a pore hosting a single Xe atom (orange arrow), a partially filled pore (violet arrow) and a fully filled pore (dark-blue arrow). (**e**–**g**) Subsequent STM images acquired during a sequence of Xe-repositioning experiments targeted at the addition of Xe atoms one by one to the same pore, demonstrating the repulsion between Xe and the confined state. The Xe-decorated tip was placed above the pore centre during each attempt to deposit Xe (yellow cross); (**f**) the first attempt of the shown sequence resulted in the migration of a Xe atom (green dot) to the pore boundary, whereas (**g**) the second in the diffusion of one of the Xe atoms to an unoccupied corner of the pore (green arrow) and in the jump of the other Xe atom to a neighbouring pore (blue dashed arrow). The black dot in **e** marks Xe adsorbed on the node of the network (Supplementary Fig. 2). All scale bars: 1 nm.

We employ a highly ordered Cu-coordinated, triply dehydrogenated 4,9-diaminoperylene quinone-3,10-diimine (3deh-DPDI) porous network grown on Cu(111)^21^,^22^ as a template. The electronic Shockley surface state of the underlying substrate is confined in the pores^23^, resulting in a single quantum well state per pore with the spatial |Ψ|^2^ maximum in its centre (Fig. 1a–c) and thus in a specific electronic environment in each pore. As a model condensate we choose Xe atoms, which provide an ideal probe of the weak interactions because of their closed-shell electronic configuration.

### Repositioning sequences of single Xe atoms

Upon deposition of Xe unto the structured surface, the pores of the network were found to host different numbers of Xe atoms, as revealed in the scanning tunnelling microscopy (STM) image shown in Fig. 1d. Notably, no single Xe atom was observed to adsorb spontaneously in the centre of a pore. To investigate whether it was possible to place a single Xe atom at this position, we performed repositioning sequences as displayed in Fig. 1e–g. In each sequence the tip was first decorated with a single Xe atom and then placed above the pore centre where its deposition was performed. Despite numerous attempts to place the Xe atom in the pore centre, none was successful. Each attempt resulted in the diffusion of the Xe atom to the border of the pore (Fig. 1f) or even in its displacement to a neighbouring pore (Fig. 1g). We assign this behaviour to the Pauli repulsion^9^ between the Xe atoms and the quantum well ground state of the pore. This contrasts with the observation made for open-shell Fe atoms and the π-acceptor CO molecules adsorbed in the pores of supramolecular networks grown on Cu(111), which experienced an attractive interaction with the quantum well state^24^,^25^.

### Spontaneously occurring Xe condensates

**Figure 2:**
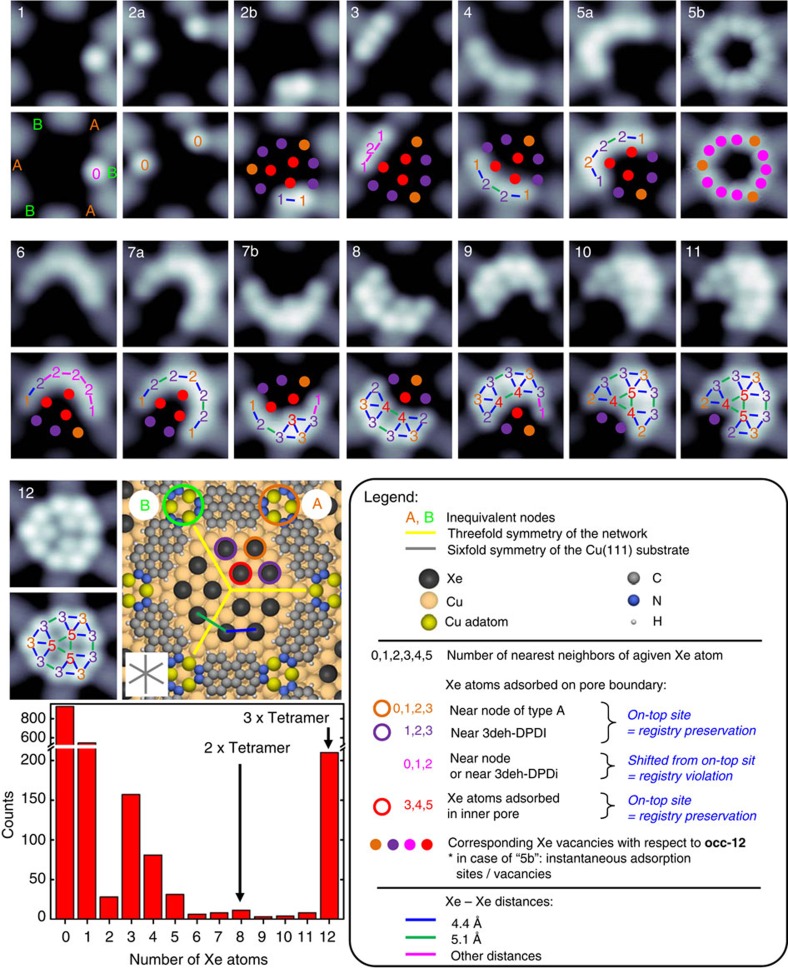
Atom-by-atom self-assembly of Xe within quantum boxes influenced by the interplay of directional weak interactions. Pores with different numbers of adsorbed Xe atoms are indicated by the numbers placed in the left upper corner of each STM image (2.4 nm × 2.4 nm); letters are used to denominate condensates with the same number of Xe atoms but having different arrangements. As a guide to the eye, a colour code that defines the adsorption position and the number of nearest neighbours is used and is defined in the legend. In the bottom left corner, the histogram of the occupancy of the pores obtained from one sample exposed to 120 L of Xe at 9 K, resulting in a coverage Θ=0.178 (*cf*. Supplementary Note 1), is displayed (2,067 pores have been analysed).

The spontaneously occurring occupancies ranging from 0 to 12 Xe atoms (denoted hereafter as **occ-0**—**occ-12**) were found across the pores of the network. Figure 2 presents the various spatial arrangements of Xe atoms, with two different forms of aggregation being observed for **occ-2**, **occ-5** and **occ-7**. The histogram of pore occupancy (Fig. 2) reveals the presence of favoured occupancies, which may be related to particularly stable condensates. The most favoured occupancies are **occ-1** and **occ-12**, whereas condensates with greater occupancies (**occ-6**—**occ-11**) were less frequently observed. We note that **occ-3** and **occ-4** occur more frequently than **occ-2,** and that there is a slight preference for **occ-8**, indicating an increased stability of multiples of a tetrameric arrangement. In contrast, the occupancy of Fe adatoms inside pores of an organic on-surface network mentioned above was found to correspond to a Poisson distribution^24^, further accentuating the fundamental differences between (open shell) metal atom condensation and noble gas condensation in somewhat related porous confinements, both of which exhibit a quantum well state (*cf*. Supplementary Fig. 1).

## Discussion

For an analysis of the self-assembled patterns of Xe atoms within the pores, a closer inspection of the metal–organic surface structure is essential. Each pore of the network possesses threefold symmetry because of the inequivalence of its nodes. The node labelled **A** in the schematic representation of an **occ-12** pore (Fig. 2) is centred above a hollow surface site, whereas the node **B** is centred above an on-top site of a surface atom^22^. Three different adsorption sites of Xe are identified: one in the inner pore (which is only occupied for **occ-7b** and larger) and two at the pore boundary either near the organic network molecule or in the vicinity of node **A**. The sites next to node **B** are never occupied in the **occ-12** condensates, and therefore the Xe adsorption reflects the threefold rotational symmetry of the pores as indicated by the three yellow lines in the schematic **occ-12** model in Fig. 2. In agreement with this, each **occ-12** condensate consists of three tetramers, with Xe atoms adsorbed in on-top sites of the Cu(111) atomic lattice in a (√3 × √3)R30° overlayer structure, in accordance with previous studies of Xe on Cu(111)^26^,^27^.

**Figure 3:**
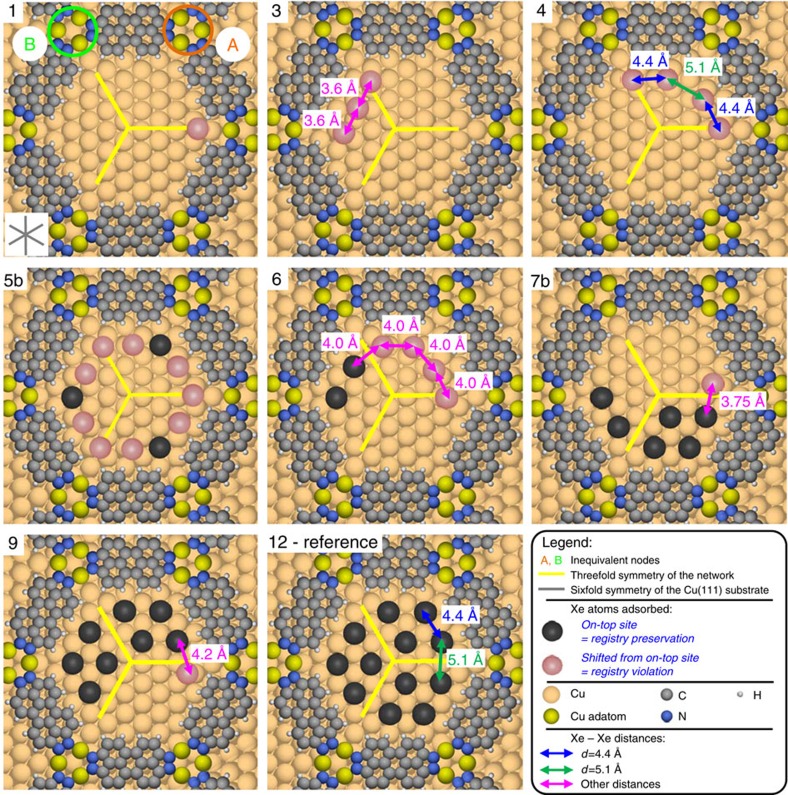
Registry violation observed in Xe self-assembly within quantum boxes. Tentative models of the **occ-*****n*** exhibiting registry violation (the **occ-*****n*** labelling is consistent with the one presented in Fig. 2. A colour code is defined in the legend). In case of **occ-1** the single Xe atom was found to be adsorbed near both types of nodes with a slight preference for the node of type **B** (57%; Table 1). As in the proximity of this type of node no on-top site is available, the adsorption site of a Xe atom in this case is shifted to the hollow site. The same phenomenon is observed for **occ-2a**. Important to note is that the distance of ~3 Å between Xe and hydrogen of 3deh-DPDI is the same in both cases (adsorption near node **A** and near node **B**), implying that the proximity with the backbone can modify the Xe adsorption site. We tentatively assign this preference of Xe to adsorb in the vicinity of node **B** to be caused by the inequality of the nodes, which leads to slightly different strengths of the Xe–H interaction. In case of **occ-3**, **occ-6**, **occ-7b** and **occ-9**, a registry violation is always observed for one, three or four Xe atoms (*cf*. Fig. 2, Table 1).

For the classification of the structural arrangements of the **occ-*****n*** inside the pores, we initially focus on two aspects: first, whether a certain *n*-mer exhibits a subset of the adsorption sites observed for the **occ-12**, meaning that the (√3 × √3)R30° structure, exhibited by each tetramer of **occ-12**, is preserved, and second, whether the considered **occ-*****n*** can be described either as **occ-(*****n*****-1)** with one additional Xe atom, or as superposition of condensate structures observed for lower occupancies. Focusing on the first aspect, we find that only condensates **occ-2b**, **occ-5a**, **occ-7a**, **occ-8**, **occ-10** and **occ-11** are always congruent with the registry of the **occ-12**. The other condensates (partially) violate the registry of the dodecamer (Figs 2 and 3, Table 1), implying that the interaction of a single Xe atom with the backbone of the organic network is sufficient to cause the Xe atom to occupy a slightly less favourable adsorption site. Moreover, the modified quantum well state may also have an impact on the favourability of the adsorption sites of Xe atoms. Furthermore, the observed registry violations retain or decrease the Xe–Xe distance, indicating an equal or increased Xe–Xe condensation energy as compared with the corresponding positions identified for **occ-12** (Figs 2 and 3).

With the second aspect brought into focus, namely the relationship between **occ-*****n*** and its predecessors in the hierarchy, our analysis shows that only condensates **occ-2**, **occ-5a**, **occ-9**, **occ-11** and **occ-12** feature the same arrangement of adsorption sites as their corresponding **occ-(*****n*****-1)** condensates. In fact, they derive from their predecessor by having one additional adsorption site occupied. Only condensates **occ-2a**, **occ-4** and **occ-7a** can be described by a superposition of condensates observed at lower occupancies. Moreover, all observed condensates were found to be stable with the exception of **occ-5b** in which five Xe atoms are sharing 12 adsorption sites along the rim of the pore and exhibit rapid site exchange (Fig. 2; the number of Xe atoms in this condensate was identified from Xe-repositioning sequences).

In summary, the atom-by-atom condensation of Xe in the pores of the Cu-coordinated 3deh-DPDI network results in a wide range of occupancies (**occ-1**—**occ-12**), which do not follow a single set of ‘hierarchic filling rules’^25^, but adapt their structures in the different regimes. This work demonstrates that the confinement of adsorbates in the pores of a metal–organic on-surface network provides the opportunity to study condensation under the influence of the subtle interplay of weak forces with single-atom precision. The experimental resolution providing the real-space structure of atomic condensates adsorbed on an atomically defined patterned surface provides the unique opportunity to compare experimental data and theoretical models. We note that our approach can benefit from the comparison of the condensation behaviour in differently sized pores, as owing to that, the interference of weak interactions involved can be tuned, which is expected to be stronger/weaker with decreasing/increasing pore size.

## Methods

### Sample preparation

The samples were prepared and investigated in an ultrahigh vacuum system with a base pressure of 5 × 10^−11 ^mbar. The Cu(111) crystal (MaTecK GmbH) was prepared by cycles of Ar^+^ sputtering at *E*=1,000 eV performed at room temperature followed by annealing at 800 K. The DPDI molecules were deposited with the use of nine-cell commercial evaporator (Kentax, GmbH, Germany) on the Cu(111) by sublimation at ~240 °C. The rate was controlled before deposition by a quartz crystal microbalance. After deposition, the sample was annealed to 300 °C in order to convert DPDI into 3deh-DPDI, which creates the Cu-coordinated network^21^. Xe of purity 99.99% was dosed to the sample placed in the STM (Omicron Nanotechnology GmbH) operated at 4.2 K, with the cryoshields open and the leak valve being in line-of-sight with the sample. Supplementary Fig. 2 presents STM data acquired after exposure of the Cu-coordinated 3deh-DPDI network to 20 L of Xe performed at a pressure equal to 1.3 × 10^−7 ^mbar for 200 s causing the increase in the sample temperature to 8 K. Xe was found to be adsorbed in the pores as well as in the nodes of the network. Figure 1d and the histogram in Fig. 2 present data after a 120-L exposure at the same pressure for 1,200 s resulting in the increase in the sample temperature to 9 K. Only Xe adsorbed in the pores was found.

### Repositioning sequences of single Xe atoms

All the self-assembled condensates, except of **occ-2b**, were reproduced with the use of repositioning sequences, by taking away Xe atoms from the 12-fold occupied pore as these are represented in Fig. 2. The difficulty in reproducing **occ-2b** can be connected with increased stability of **occ-3**, as revealed by the histogram of pore occupancy (Fig. 2). To perform Xe manipulations in a reproducible manner, a home-written Labview software compatible with the Nanonis SPM control system (Specs GmbH) was used. To pick up a single Xe atom, in the first step the sample bias was set to −2 mV and the tip was moved towards the atom until a not-continuous change in the resistance of the tunnelling junction occurred. Then the tip was retracted. To place a Xe atom in a desired place, the sample bias was set to 800 mV and the procedure was repeated.

### STM measurements and data analysis

STM measurements were performed in the constant current mode with Pt–Ir tips (90% Pt, 10% Ir), prepared by mechanical cutting followed by sputtering and controlled indentation in the bare Cu(111). The STM images shown in Fig. 1a,b were acquired with such prepared metallic tip, whereas the others with additionally Xe-decorated tips, which allowed achieving atomic resolution on Xe condensates (Supplementary Fig. 3). The STM image with the simultaneously acquired d*I*/d*V* map, shown in Fig. 1a,b, was measured with −200 mV/200 pA and with a lock-in frequency of 512 Hz and a zero-to-peak value of 8 mV. To avoid modification of the condensates via interaction with the tip, the sample bias was selected within a range of −10 to −80 mV, whereas the tunnelling current was set within the range 5–50 pA. The exact tunnelling parameters of STM images presented in the main text are as follows: −10 mV/10 pA (Fig. 2, **occ-3**, **occ-5b**, **occ-9**, **occ-10**), −10 mV/20 pA (Fig. 2, **occ-11**), −10 mV/50 pA (Fig. 2, **occ-1**, **occ-8**, **occ-12**), −20 mV/5 pA (Fig. 2, **occ-7b**), −20 mV/10 pA (Fig. 2, **occ-2a**), −50 mV/5 pA (Fig. 2, **occ-6**), −50 mV/10 pA (Figs 1d and 2, **occ-2b**, **occ-4**, **occ-7a**), −80 mV/5 pA (Fig. 2, **occ-5a**), −200 mV/80 pA (Fig. 1e) and −500 mV/50 pA (Fig. 1f,g). The STM data were processed with the WSxM software^28^. For better comparability of the data, the colour histograms have been adjusted. Low-pass filtering was used for noise reduction.

## Author contributions

S.N., A.W., S.K., T.I., J.N., S.F., C.W. and T.N conducted the STM measurements and analysed the data under the supervision of T.A.J., L.H.G., M.S. and E.M.; J.B. and T.I. provided theoretical models; S.N., T.A.J. and L.H.G. wrote the manuscript.

## Additional information

**How to cite this article:** Nowakowska, S. *et al*. Interplay of weak interactions in the atom-by-atom condensation of xenon within quantum boxes. *Nat. Commun.* 6:6071 doi: 10.1038/ncomms7071 (2015).

## Supplementary Material

Supplementary Information Supplementary Figures 1-3, Supplementary Note, Supplementary Reference

## Figures and Tables

**Table 1:** Condensation regimes of Xe adsorbed in the pores of the Cu-coordinated 3deh-DPDI network.
