# Supplementary material for: Interplay of weak interactions in the atom-by-atom condensation of xenon within quantum boxes
Source: Nat Commun. 2015 Jan 21;6:6071. doi: 10.1038/ncomms7071 (PMC4354259; doi:10.1038/ncomms7071)
Supplement: Supplementary Information — Supplementary Figures 1-3, Supplementary Note, Supplementary Reference [file ncomms7071-s1.pdf]

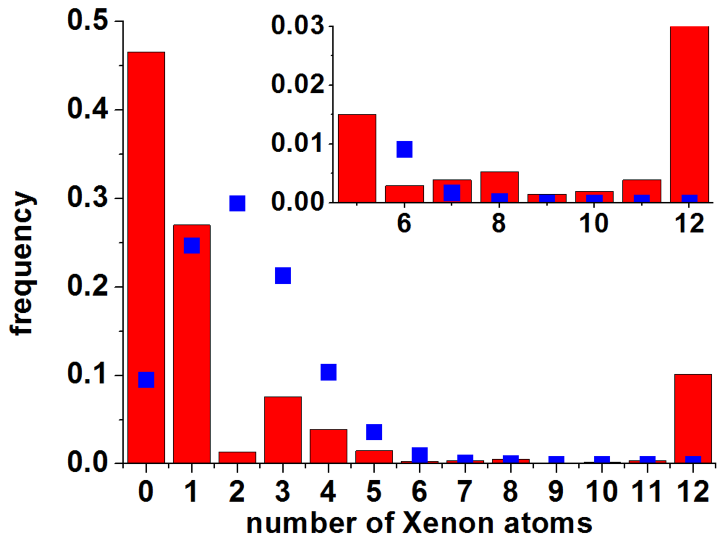

Supplementary Figure 1 | The comparison of the frequency histogram of the occupancy of the pores obtained from the sample exposed to 120 L of Xe at 9 K, resulting in a coverage of Θ=0.178 (red bars), with the binomial distribution for this coverage value (blue squares). Clearly, **occ-*n*** do not follow a statistical distribution.

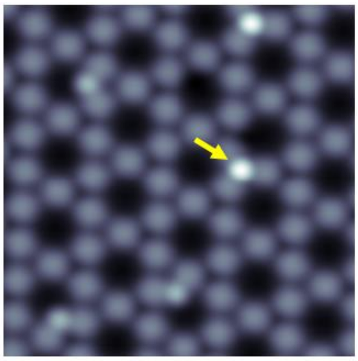

Supplementary Figure 2 | Adsorption of Xe on the nodes and in the pores of the Cu-coordinated 3deh-DPDI network; STM image of the network after exposure to 20 L of Xe during which the sample temperature did not exceed 8 K. In addition to Xe adsorbed in the pores, single Xe atoms were found to adsorb on the nodes of the network as indicated by the yellow arrow (10 nm x 10 nm; tunneling parameters: -1 V/10 pA; metallic tip). On the contrary, after Xe exposure at higher sample temperature (9 K) Xe was only found to be adsorbed in the pores, which points at a temperature dependent site selectivity of Xe adsorption.

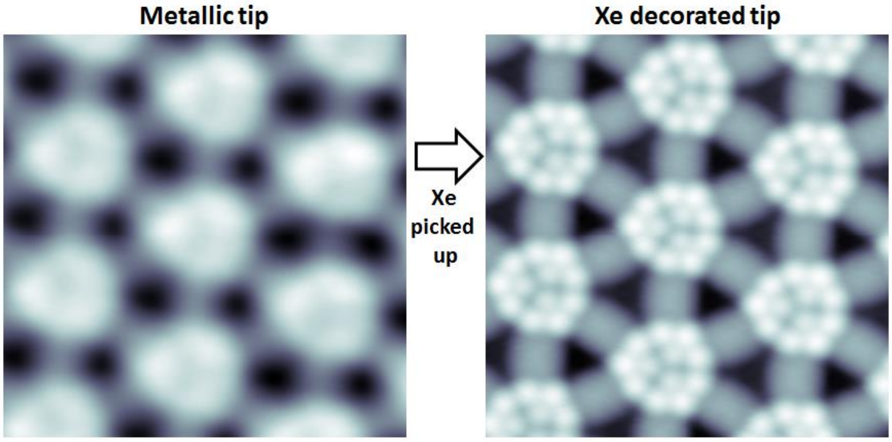

Supplementary Figure 3 | Comparison of the STM contrast of Xe condensates acquired with a metallic and a Xe decorated tip (6 nm x 6 nm; tunneling parameters: 10 mV/50 pA). By warming up the sample (Cu-coordinated 3deh-DPDI on Cu(111) + 120 L of Xe) to 45 K followed by cooling to 5 K for STM measurements all pores could be filled with 12 Xe atoms due to the diffusion of Xe from the islands grown around the network domains. This is in line with the study of Park et al., [Ref.1] who reported that above 25 K the diffusion of Xe on Cu(111) occurs. Moreover, Xe atoms have been employed for a functionalization of STM tips for high resolution imaging.[2]

Supplementary Note 1

The coverage (Θ) of Xe in the pores of the Cu-coordinated 3-deh DPDI network was calculated in a following way:

$$\Theta = \frac{N}{a \cdot n}$$

N – total number of Xe atoms adsorbed in the pores;

a – number of adsorption sites per one pore;

n – total number of pores;

The binomial distribution, determined according to Ref. [3], for the Θ=0.178 (sample exposed to 120 L of Xe at 9 K) is displayed in Fig. 2 together with the experimentally obtained frequency histogram. Clearly, the **occ-*n*** do not follow a binomial distribution, which means that Xe atoms are able to diffuse from pore to pore before being cooled down to 4.2 K for the STM measurements.

Supplementary References

1 Park, J.-Y. *et al.* Adsorption and growth of Xe adlayers on the Cu(111) surface. *Phys. Rev. B* **60,** 16934–16940 (1999).

2 Kichin, G., Weiss, C., Wagner, C., Tautz, F. S. & Temirov, R. Single Molecule and Single Atom Sensors for Atomic Resolution Imaging of Chemically Complex Surfaces. *J. Am. Chem. Soc.* **133,** 16847–16851 (2011).

3 Pivetta, M., Pacchioni, G. E., Schlickum, U., Barth, J. V. & Brune, H. Formation of Fe Cluster Superlattice in a Metal-Organic Quantum-Box Network. *Phys. Rev. Lett.* **110**, 086102 (2013).
